# Supplementary material for: The interactome of intact mitochondria by cross-linking mass spectrometry provides evidence for coexisting respiratory supercomplexes
Source: Mol Cell Proteomics. 2017 Dec 8;17(2):216–32. doi: 10.1074/mcp.RA117.000470 (PMC5795388; doi:10.1074/mcp.RA117.000470)
Supplement: Supplemental Data [file supp_RA117.000470_133922_0_supp_23978_fzffwf.zip › spectra_annotation/mito_spectra_annotation/164-1-4-1-4-1.pdf]

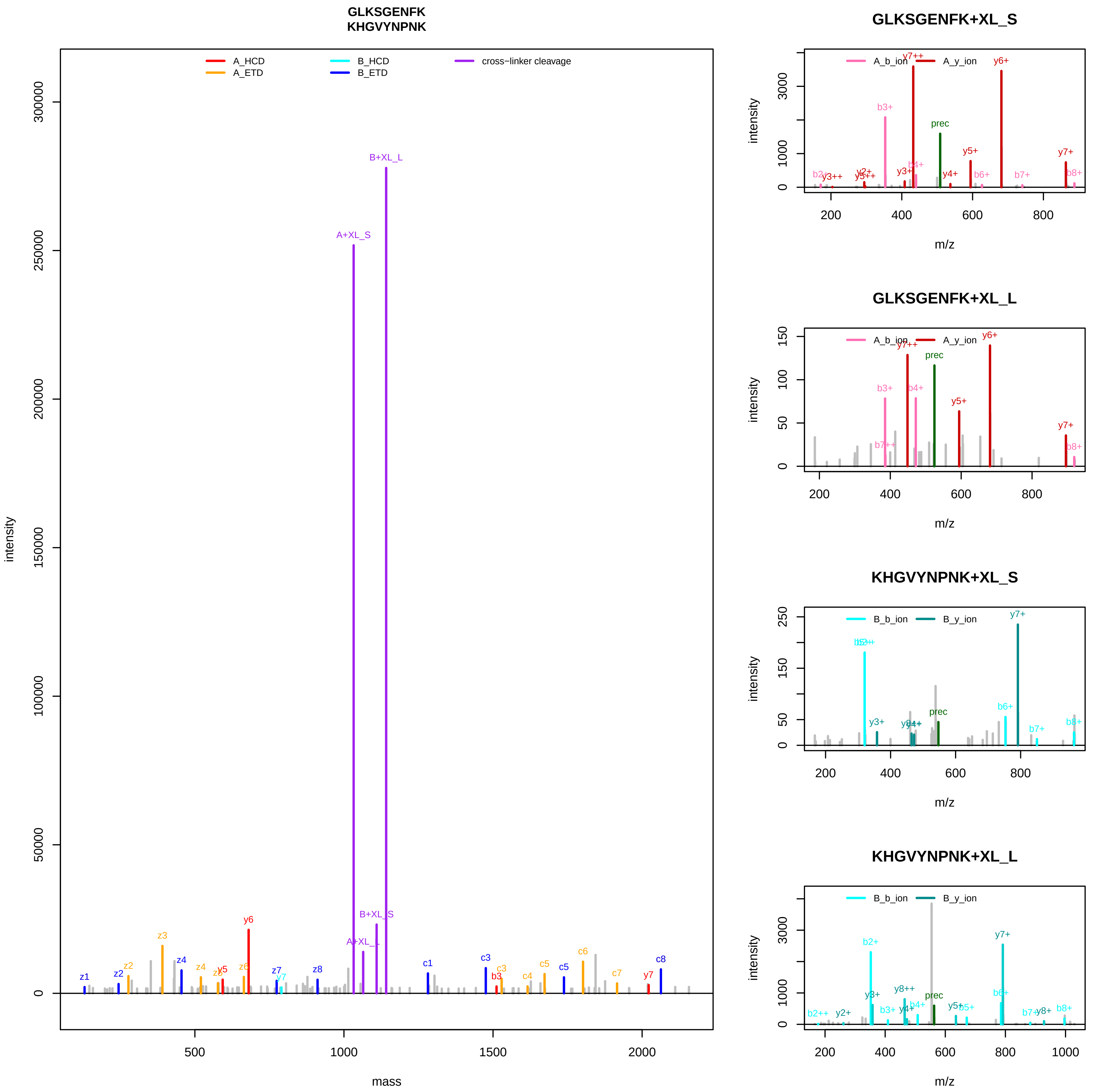
GLKSGENFK
KHGVYNPNK
A_HCD
A_ETD
B_HCD
B_ETD
cross−linker cleavage
B+XL_L
A+XL_S
B+XL_S
A+XL_L
y6
y5
y7
z1
z2
z3
z4
z5
z6
z7
z8
c1
c3
c4
c5
c6
c7
c8
b3
intensity
mass
0
50000
100000
150000
200000
250000
300000
500
1000
1500
2000
GLKSGENFK+XL_S
GLKSGENFK+XL_L
KHGVYNPNK+XL_S
KHGVYNPNK+XL_L
A_b_ion
A_y_ion
B_b_ion
B_y_ion
prec
m/z
